# Can flaxseed “milk” prevent anthracycline mediated cardiotoxicity in women with breast cancer (CANFLAX-BC)?

**DOI:** 10.1186/s40959-025-00441-3

**Published:** 2026-01-08

**Authors:** Vibhuti Arya, Lana Mackic, Sara M. Telles Langdon, David Y. C. Cheung, Paris R. Haasbeek, Skyler Eastman, Lauren Castagna, Scott Grandy, Stefan S. Heinze, Danielle Desautels, Vallerie Gordon, Jeffrey Graham, Susan Green, Debjani Grenier, Christina A. Kim, Maclean Thiessen, Marshall Pitz, Davinder S. Jassal

**Affiliations:** 1 Institute of Cardiovascular Sciences, St. Boniface Albrechtsen Research Centre, University of Manitoba, Winnipeg, MB Canada; 2 Department of Physiology and Pathophysiology, Rady Faculty of Health Sciences, Max Rady College of Medicine, University of Manitoba, Winnipeg, MB Canada; 3 Faculty of Science, University of Manitoba, Winnipeg, MB Canada; 4 Faculty of Health, School of Health and Human Performance, Dalhousie University, Winnipeg, MB Canada; 5 Faculty of Medicine, Dalhousie University, Winnipeg, MB Canada; 6 Department of Internal Medicine, Section of Medical Oncology and Hematology, University of Manitoba, Winnipeg, MB Canada; 7 Department of Internal Medicine, Rady Faculty of Health Sciences, Section of Cardiology, Max Rady College of Medicine, University of Manitoba, Winnipeg, MB Canada; 8 Department of Radiology, Rady Faculty of Health Sciences, Max Rady College of Medicine, University of Manitoba, Winnipeg, MB Canada; 9 Department of Internal Medicine, Rady Faculty of Health Sciences, Section of Cardiology, Bergen Cardiac Care Centre, Max Rady College of Medicine, University of Manitoba, St. Boniface Hospital, 409 Tache Avenue, Winnipeg, MB R2H 2A6 Canada

**Keywords:** Cardio-Oncology, Flaxseed, Nutraceutical, Anthracyclines, Echocardiography, Breast cancer, Heart failure

## Abstract

**Background:**

Cardio-Oncology focuses on the prevention, diagnosis, and management of individuals with cancer who are at risk of developing cardiovascular complications as a result of their anti-cancer treatment. The aim of the “*Can flaxseed “milk” prevent chemotherapy mediated cardiotoxicity in women with breast cancer* ***(****CANFLAX-BC) study*” was to investigate whether consumption of flaxseed (FLX) “milk” can prevent cardiac dysfunction in women with breast cancer treated with anthracyclines.

**Methods:**

In this small pilot double-blinded, single centre, prospective randomized controlled clinical trial, women with breast cancer were randomized to oral consumption of either placebo oat fiber “milk” or FLX “milk” for a total of 4 months while receiving anthracycline-based chemotherapy. Serial echocardiography and cardiac biomarkers were measured at baseline, 4-months, and 6-months follow-up.

**Results:**

Between 2021 and 2023, a total of 21 women with early-stage breast cancer (mean age 48 ± 10 years, BMI 28 ± 5 kg/m^2^), treated with an anthracycline based chemotherapy regimen, were enrolled in the CANFLAX-BC study. During the 4-month intake period, a total of 8 women received oat fibre “milk” and 13 women received FLX “milk” while receiving anthracyclines, with a comparable adherence rate of 70% for both groups. At baseline, 4-months, and 6-months follow-up, the LVEF was 68±4%, 69±3%, and 66±4%, respectively, for the oat fibre “milk” group and 67±5%, 68±4%, and 66±5%, respectively, for the FLX “milk” group (p = NS). Although the mean global longitudinal strain (GLS) was comparable between both groups at baseline, the GLS was lower at -13.8 ± 0.3% (4 months) and − 15.1 ± 0.3% (6 months) for the oat fibre “milk” group as compared to a GLS of -18.4 ± 0.3% (4 months) and − 18.5 ± 0.2% (6 months) for the FLX “milk group (p < 0.05).

**Conclusion:**

In women receiving an anthracycline based chemotherapy regimen for breast cancer, this small pilot study suggests that FLX “milk” consumption may prevent early cardiotoxicity as reflected by preservation of GLS parameters.

## Introduction

Cardiovascular disease and cancer are the leading causes of morbidity and mortality in Canada (https://www.heartandstroke.ca/articles/connected-by-the-numbers) (https://cancer.ca/en/research/cancer-statistics/cancer-statistics-at-a-glance). These two diseases are intricately linked, as the treatment of cancer may cause detrimental effects to the heart. Although anthracyclines, including Doxorubicin (DOX) and Epirubicin (EPI), improve overall survival in women with breast cancer, the clinical use of these agents is limited by their cumulative dose-dependent cardiotoxicities [1–5]. Amongst the various pharmacological therapies which may prevent anthracycline mediated cardiotoxicity including anti-oxidants, renin-angiotensin system (RAS) antagonists, and beta-blockers [6–12], the use of nutraceuticals, specifically flaxseed (FLX), is novel and has not been studied to date in the clinical setting.

Flaxseed, in addition to its basic nutritional function, has a positive health effect in several disease conditions, including cancer and cardiovascular disease [13]. Flaxseed is an oilseed that is rich in both α-linolenic acid (ALA) and the lignan secoisolariciresinol diglucoside (SDG), which have potent anti-inflammatory and anti-oxidative properties, respectively [13]. The FLAX-PAD study previously demonstrated the health benefits of consuming 30 g of FLX daily (FLX containing food products including muffins, bagels, snack bars, and milled seeds) in the management of hypertension and peripheral vascular disease (PVD) [14]. A number of recent meta-analyses have also confirmed that consumption of 30 g of FLX daily prevents breast cancer development and recurrence [15–19]. In a pre-clinical model of anthracycline mediated cardiotoxicity, we demonstrated the cardioprotective role of FLX in preventing adverse cardiovascular remodeling by attenuating inflammation, apoptosis, oxidative stress, and mitochondrial dysfunction [20, 21]. Little is known, however, on whether FLX is cardioprotective in the prevention of anthracycline-mediated cardiotoxicity in the clinical setting.

The objective of the “*Can flaxseed “milk” prevent chemotherapy mediated cardiotoxicity in women with breast cancer* ***(****CANFLAX-BC) study*” was to investigate whether consumption of FLX “milk” can prevent cardiac dysfunction in women with breast cancer treated with anthracyclines.

## Methods

Between September 2021 and December 2023, a total of 21 consecutive female patients with breast cancer were prospectively recruited from the oncology clinics located at the two major CancerCare Manitoba sites including St. Boniface Hospital and Health Sciences Centre in Winnipeg, Manitoba, Canada. The inclusion criteria included: 1) > 18 years of age; 2) diagnosed with breast cancer (stages I-IIIA) but have not started chemotherapy; 3) scheduled to receive AC (doxorubicin and cyclophosphamide) or FEC (fluorouracil, epirubicin, and cyclophosphamide) based chemotherapy; and 4) no concurrent consumption of FLX. Any patient who was receiving RAS antagonists and/or beta-blockers or had a pre-existing diagnosis of heart failure with LVEF < 40% was excluded from the study. The study protocol was approved by the local institutional review board [University of Manitoba REB: HS22437 (B2018:134)].

Baseline demographic data was collected using CancerCare Manitoba’s electronic medical record database (ARIA). All study participants were randomly assigned in a double-blind fashion to 2 servings (660 mL; 30 g) of either placebo oat fibre “milk” or FLX “milk” daily for a total of 4 months while receiving and completing their anthracycline-based chemotherapy. The composition of both oat fibre “milk” and FLX “milk” was nearly identical with common ingredients including omega-3 fatty acids, natural vanilla flavoring, cane sugar, gums for texture, Vitamin A palmitate, Vitamin D2, Vitamin B12, salt, and water. Each serving (330 mL) of the oat fibre “milk” contained 15 g of oats and the FLX “milk” contained 15 g of milled whole FLX. Although the previous FLAX-PAD study demonstrated the health benefits of consuming 30 g of FLX daily (FLX containing food products including muffins, bagels, snack bars, and milled seeds) in the management of hypertension and PVD [14], we used a FLX “milk” product for the current study to improve overall compliance for the CANFLAX study. In total, the patient population was evaluated at 3 separate time points: (i) before the initiation of anthracycline-based chemotherapy; (ii) 4-months; and ii) 6-months after initiation of chemotherapy.

At each visit, blood pressure was recorded in each study participant and blood was drawn to measure plasma ALA levels and N-terminal pro-B-type natriuretic peptide (NT-proBNP). Serial transthoracic echocardiographic (TTE) studies were also performed at each time point. Each study participant kept a consumption log and were contacted weekly to evaluate “milk” consumption and determine assigned treatment compliance. As an objective compliance measure, gas chromatography-flame ionization was used to quantify plasma ALA levels which served as a surrogate of FLX “milk” consumption. Plasma NT-proBNP was measured at three separate timepoints using a sandwich enzyme-linked immunosorbent assay with neat plasma samples as per the manufacturer’s instruction (catalogue #KA3099; Abnova). Finally, serial TTE was performed on a GE Vivid IQ platform (GE Medical Systems, Milwaukee, Wisconsin, US). Left ventricle (LV) cavity dimensions and LV ejection fraction (LVEF) were determined from 2-dimensional images as per the American Society of Echocardiography (ASE) guidelines [22]. Global longitudinal strain (GLS) was calculated using automated function imaging (AFI) from the apical long-axis, 4-chamber, and 2-chamber views on the GE Healthcare’s EchoPAC software. The echocardiographic studies were examined in a blinded fashion.

The data are summarized as mean ± standard deviation, number (percentage), or median and interquartile range. Chi-square and Fisher exact tests were applied to compare categorical data. Comparison of variables within each group versus baseline was performed with repeated measures analysis of variance and Dunnett’s test. Cancer therapy-related cardiac dysfunction (CTRCD) was defined as a significant decrease in LVEF > 10% when compared to baseline or an absolute LVEF value < 53% [23]. A significant decrease in GLS was defined as a > 15% relative reduction from baseline as per established guidelines [23]. A P-value of < 0.05 was considered significant. The Statistical Analysis System (version 9.4, SAS Institute, Cary, North Carolina) was used to perform the analysis.

## Results

A total of 21 women with early-stage breast cancer (EBC) (mean age 48 ± 10 years, BMI 28 ± 5 kg/m^2^), treated with an anthracycline based chemotherapy regimen, were enrolled in the CANFLAX-BC study. A total of 8 women were randomized to the oat fibre “milk” group and 13 women to the FLX “milk” group for a total of 4 months while receiving and completing their anthracycline-based chemotherapy. The prevalence of underlying cardiovascular risk factors was low in the study population; except for a higher incidence of a family history of premature coronary artery disease in the FLX “milk” group (Table 1). There was no difference in the location, grade, and stage of the underlying breast cancer between both groups (Table 1). The majority of patients (86%) received 4 cycles of doxorubicin and cyclophosphamide (AC) therapy, whereas the remaining 14% received 3 cycles of 5-fluorouracil, epirubicin, and cyclophasmide and 3 cycles of docetaxel (FEC-D) (Table 1).

**Table 1:** Baseline demographic data of study participants

Baseline Characteristics	Oat fibre “milk” (*n* = 8)	FLX “milk” (*n* = 13)	Total (*n* = 21)	*P*-values
Age (years) (mean ± SD)	48 ± 11	47 ± 10	48 ± 10	0.83
BMI (kg/m^2^) (mean ± SD)	27 ± 4	29 ± 5	28 ± 5	0.34
Cardiovascular Risk Factors				
Hypertension (*n*, %)	1 (13%)	0 (0%)	1 (5%)	0.80
Hyperlipidemia (*n*, %)	0 (0%)	1 (8%)	1 (5%)	1.00
Diabetes (*n*, %)	0 (0%)	1 (8%)	1 (5%)	1.00
Smoking history (*n*, %)	1 (13%)	1 (8%)	2 (10%)	1.00
Family history of CAD (*n*, %)	1 (13%)	6 (46%)	7 (33%)	0.27
Features of Cancer				
Estrogen receptor positive (*n*, %)	7 (88%)	8 (62%)	15 (71%)	0.43
Progesterone receptor positive (*n*, %)	7 (88%)	8 (62%)	15 (71%)	0.43
HER2 positive (*n*, %)	1 (13%)	3 (23%)	4 (19%)	0.98
Location of cancer – right only (*n*, %)	3 (38%)	6 (46%)	9 (43%)	1.00
Location of cancer – left only (*n*, %)	5 (63%)	7 (54%)	12 (57%)	1.00
				N/A
Lymph node positive (*n*, %)	7 (88%)	13 (100%)	20 (95%)	0.80
Grade 1 carcinoma (*n*, %)	1 (13%)	1 (8%)	2 (10%)	1.00
Grade 2 carcinoma (*n*, %)	4 (50%)	6 (46%)	10 (48%)	1.00
Grade 3 carcinoma (*n*, %)	3 (38%)	6 (46%)	9 (43%)	1.00
Stage 1 carcinoma (*n*, %)	0 (0%)	3 (23%)	3 (14%)	0.41
Stage 2 carcinoma (*n*, %)	4 (50%)	5 (39%)	9 (43%)	0.95
Stage 3 carcinoma (*n*, %)	4 (50%)	5 (39%)	9 (43%)	0.95
Cancer Treatment				
Mastectomy (*n*, %)	2 (25%)	6 (46%)	8 (38%)	0.61
Lumpectomy (*n*, %)	1 (13%)	2 (15%)	3 (14%)	1.00
Radiation (*n*, %)	0 (0%)	4 (31%)	4 (19%)	0.24
Adjuvant chemotherapy (*n*, %)	3 (33%)	6 (46%)	9 (43%)	1.00
Neoadjuvant chemotherapy (*n*, %)	5 (63%)	7 (54%)	12 (57%)	1.00
Chemotherapy – AC (*n*, %)	6 (75%)	12 (92%)	18 (86%)	0.65
Chemotherapy – FECD (*n*, %)	2 (25%)	1 (8%)	3 (14%)	0.65

Baseline characteristics of oat fibre “milk” (*n* = 8) and FLX “milk” (*n* = 13)

*SD* standard deviation, *BMI* body mass index, *CAD* coronary artery disease, *HER2* human epidermal growth factor receptor 2, *AC* Adriamycin, cyclophosphamide, *FEC* 5-fluorouracil, epirubicin, cyclophosphamide, and docetaxel

**Fig. 1:**
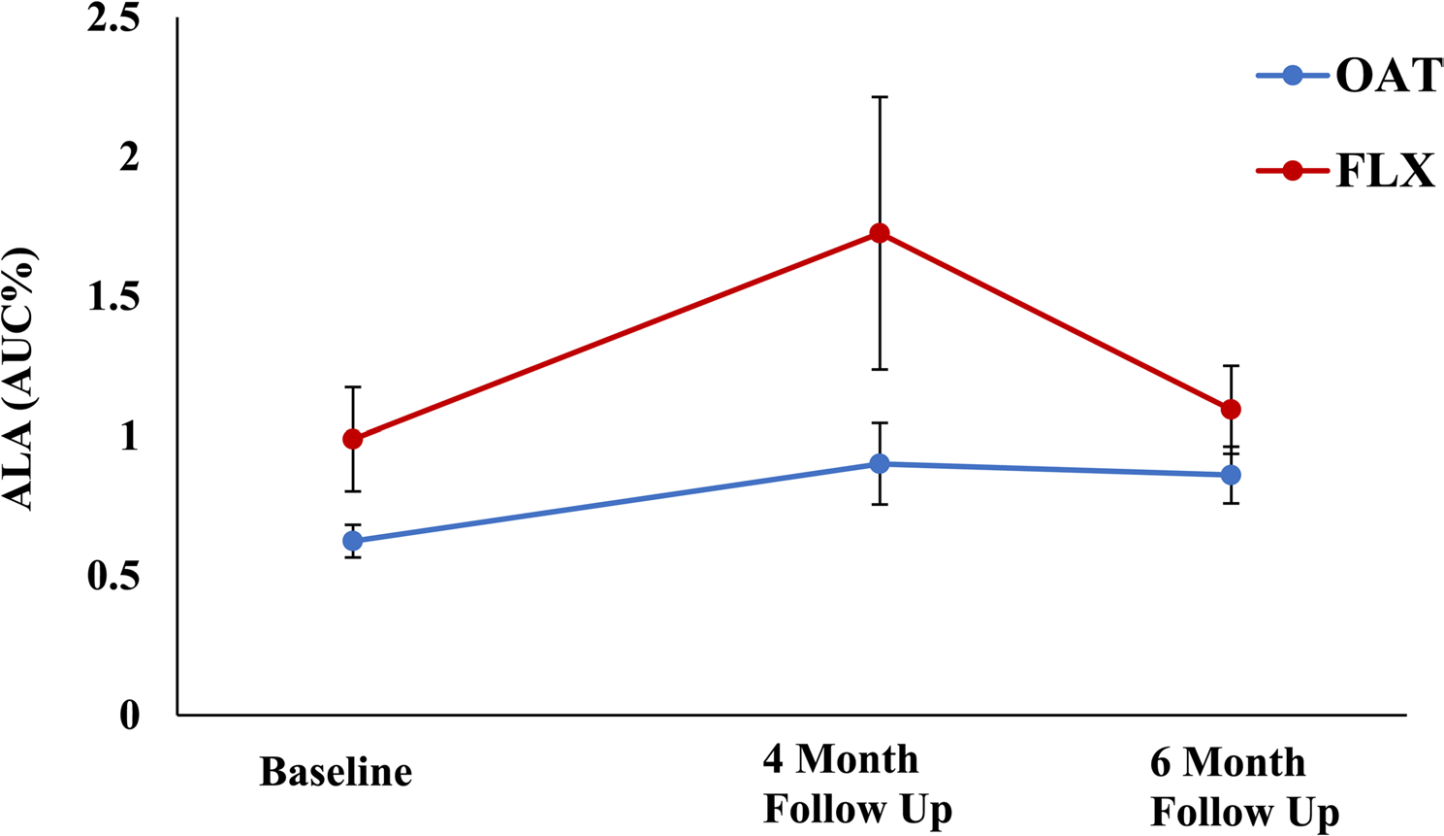
Serial plasma ALA levels

Amongst the study participants, there was an average “milk” intake adherence rate of approximately 70% during the 16-week “milk” intake period. The average total “milk” intake within the oat fibre group was 152 “milk” cartons (adherence of 68% ± 28) as compared to the FLX group which was 170 “milk” cartons (adherence of 76% ± 24). Up to 30% of study participants reported bloating due to high fibre content, development of taste aversion over time, and some difficulty maintaining a consistent intake of two bottles per day over the 4 months of treatment as major barriers to persistent “milk” intake; there were no drop outs, however, in the study population. To confirm the amount of “milk” intake in both study groups, plasma ALA concentration (marker of FLX consumption) was quantified. Comparing plasma ALA levels at baseline and 4-months follow-up, there was a 2-fold increase in the FLX “milk” group as compared to the oat fibre “milk” group (Fig. 1). At baseline, 4-months, and 6-months, the mean BP was 80 ± 4 mm Hg, 79 ± 3 mm Hg, and 81 ± 5 mm Hg in the oat “fibre” group and 82 ± 3 mm Hg, 81 ± 3 mm Hg, and 79 ± 5 mm Hg in the FLX “milk” group, respectively (no significant difference). At baseline, NT-proBNP levels were within normal limits for the entire population (Table 2). At 4-months and 6-months of follow-up, there was no significant change in the NT-proBNP levels between the oat fibre “milk” and FLX “milk” groups.

**Table 2:** Serial cardiac biomarker results

NT Pro-BNP (pmol/L)	Oat fibre “milk” (*n* = 8)	FLX “milk” (*n* = 13)	*P* value
Baseline	23.9 ± 2.1	24.4 ± 1.7	0.85
4-months	24.2 ± 1.9	25.1 ± 0.8	0.77
6-months	25.4 ± 2.5	24.9 ± 2.2	0.79

Transthoracic echocardiographic assessment of LV and RV cavity dimensions, LA volume, diastolic indices, tissue Doppler imaging parameters, and global longitudinal strain (GLS) parameters were interpreted in all 21 study participants (Table 3). There was no significant difference in these echocardiographic parameters between the oat fibre “milk” and the FLX “milk” groups. At baseline, 4-months, and 6-months follow-up, the LVEF was 68±4%, 69±3%, and 66±4%, respectively, for the oat fibre “milk” group and 67±5%, 68±4%, and 66±5%, respectively, for the FLX “milk” group (p = NS) (Table 3). Although the mean GLS was comparable between both groups at baseline, the GLS was lower at −13.8 ± 0.3% in the oat fibre “milk” group as compared to −18.4 ± 0.3% in the FLX “milk” group; this follows completion of 4-months intake of either “milk” product during concomitant treatment of their anthracycline-based chemotherapy regimen. Additionally, although the GLS at 6 months started to normalize at −15.1 ± 0.3% in the oat fibre “milk” group (despite completion of the “milk” product and chemotherapy regimen at 4 months), it was still lower than the GLS at 6 months of −18.5 ± 0.2% for the FLX “milk” group.

**Table 3:** Serial echocardiographic parameters

Echo Parameters	OAT Baseline (*n* = 8)	FLX Baseline (*n* = 13)	*P*-values	OAT 4 Months (*n* = 8)	FLX 4 Months (*n* = 13)	*P*-values	OAT 6 Months (*n* = 8)	FLX 6 Months (*n* = 13)	*P*-values
Cardiac chamber dimensions and functions									
Heart Rate (beats/min)	72±5	72±3	0.82	72±4	77±5	0.68	74±6	78±3	0.86
LVEDD (cm)	4.8±0.3	4.8±0.2	0.94	4.8±0.2	4.7±0.4	0.86	4.9±0.2	4.8±0.4	0.88
LVESD (cm)	3.1±0.2	3.0±0.3	0.85	3.2±0.2	3.0±0.3	0.85	3.2±0.1	3.2±0.3	0.84
IVS (cm)	0.9±0.1	1.0±0.1	0.85	0.9±0.1	0.9±0.2	0.94	0.9±0.2	0.9±012	0.94
PWT (cm)	0.9±0.1	1.0±0.1	0.85	0.9±0.1	0.9±0.2	0.94	0.9±0.1	0.9±0.2	0.84
LA volume (mL/m^2^)	30±3	29±2	0.78	29±4	30±2	0.75	30±3	29±4	0.81
RVEDD (cm)	2.7±0.4	2.7±0.3	0.88	2.7±0.3	2.8±0.4	0.62	2.7±0.3	2.8±0.3	0.84
LVEF (%) (Biplane Simpson’s)	68±4	67±5	0.88	69±3	68±4	0.87	66±4	66±5	097
Diastolic parameters									
E (m/s)	0.7±0.2	0.8±0.1	0.58	0.7±0.2	0.7±0.3	0.91	0.8±0.2	0.7±0.3	0.91
A (m/s)	0.5±0.2	0.6±0.1	0.48	0.5±0.1	0.5±0.2	0.88	0.5±0.1	0.6±0.2	0.81
Deceleration Time (ms)	225±8	230±6	0.38	232±5	238±7	0.42	234±5	230±7	0.58
Medial									
S’ (cm/s)	9.2±0.3	9.1±0.2	0.68	9.0±0.3	9.1±0.4	0.78	9.0±0.3	9.1±0.4	0.78
E’ (cm/s)	8.1±0.2	8.2±0.3	0.72	8.2±0.3	8.3±0.2	0.67	8.2±0.3	8.1±0.2	0.72
A’ (cm/s)	7.9±0.4	8.0±0.3	0.66	8.0±0.2	8.1±0.3	0.74	8.0±0.2	7.9±0.3	0.78
Lateral									
S’ (cm/s)	9.0±0.3	9.2±0.2	0.56	9.1±0.2	9.2±0.3	0.81	9.1±0.2	9.2±0.3	0.81
E’ (cm/s)	8.3±0.2	8.4±0.2	0.81	8.3±0.3	8.5±0.2	0.52	8.4±0.3	8.5±0.2	0.76
A’ (cm/s)	8.1±0.3	8.0±0.3	0.77	8.1±0.2	8.0±0.3	0.81	8.2±0.2	8.0±0.3	0.80
Global Longitudinal Strain									
Average GLS (%)	−18.2±0.3	−18.1±0.4	0.81	−13.8 ± 0.3	−18.4 ± 0.3	(*p* < 0.05) ^*#^	−15.1 ± 0.3	−18.5 ± 0.2	(*p* < 0.05) ^*#^

Echocardiographic parameters measured in oat fibre “milk” (*n* = 8) and FLX “milk” (*n* = 13) participants for the parasternal long axis, apical 4-chamber, and apical 2-chamber views. Data are mean ± SD at baseline and at 4-month follow-up

*LVEDD* left ventricular end diastolic diameter, *LVESD* left ventricular end systolic diameter, *IVS* interventricular septal thickness, *PWT* posterior wall thickness, *LA* left atrium, *RVEDD* right ventricular end-diastolic diameter, *LVEF* left ventricular ejection fraction, *E* early filling velocity; A, atrial filling velocity; Medial (right) and Lateral (left) mitral annulus velocity; S’, positive systolic wave velocity; E’, Early diastolic myocardial relaxation velocity; A’, Late diastolic atrial contraction velocity, *GLS* global longitudinal strain

## Discussion

Breast cancer is the leading cause of cancer-related morbidity and mortality in North America (https://www.heartandstroke.ca/articles/connected-by-the-numbers) (https://cancer.ca/en/research/cancer-statistics/cancer-statistics-at-a-glance). Although cancer therapeutics, including anthracyclines, are associated with clear therapeutic benefits in reducing overall morbidity and mortality in the breast cancer setting[1–5], increased cardiotoxicity remains a serious concern. In lieu of pharmacological agents, including RAS antagonists and beta-blockers, which have demonstrated inconclusive benefits in the prevention of CTRCD[6–12], the prophylactic use of a nutraceutical agent such as FLX is novel and has not been investigated to date. In the pre-clinical setting, we have previously demonstrated the efficacy of FLX as a cardioprotective agent in the prevention of DOX-mediated cardiotoxicity using *an in vivo* murine model [20]. – [21] The CANFLAX study is a small pilot RCT that attempts to translate our basic science findings by highlighting the consumption of FLX “milk” as a potential cardioprotective agent. With an overall “milk” adherence rate of 70% and a 2-fold increase in the ALA plasma levels, the CANFLAX study demonstrates the feasibility of consuming 30 g of FLX during concomitant treatment with an anthracycline based chemotherapeutic regimen. Although there was no change in blood pressure, cardiac biomarkers, nor LVEF in the two study groups, the preservation of GLS parameters at 4 months in the FLX “milk” group signals the potential cardioprotective role of this nutraceutical agent in women treated with anthracyclines for breast cancer.

As the prophylactic role of RAS antagonists and beta blockers in the prevention of CTRCD remains inconclusive[6–12], consensus guidelines do not currently endorse their routine use in women with breast cancer. In lieu of RAS antagonists and beta blockers, which are associated with undesirable side effects of hypotension, cough, hyperkalemia, fatigue, and/or depression, the use of nutraceuticals as an alternative cardioprotective intervention is novel in the breast cancer population. Previous clinical studies have focused on the therapeutic role of nutraceutical interventions using FLX in cardiovascular disease and breast cancer. The FLAX-PAD study investigated the effects of daily ingestion of FLX on blood pressure in a total of 110 participants with pre-existing peripheral artery disease (PAD) [14]. The consumption of 30 g of milled FLX per day in the form of various food products (muffins, bagels, snack bars, pasta, and/or buns) resulted in a significant reduction in systolic blood pressure (10 mmHg) and diastolic blood pressure (7 mmHg) in the study population [14]. These results highlight the vascular relaxation effects of FLX as a potent anti-hypertensive agent. Furthermore, supplementation of up to 30 g of FLX in diets of healthy volunteers improved lipid profiles by reducing total cholesterol levels by 6% and LDL-C by 9%[24]. Finally, a number of studies have also confirmed that the daily consumption of 30 g of FLX prevents the development of breast cancer and its recurrence by nearly 25%[15–19]. Little is known, however, on the potential cardioprotective role of consuming 30 g of FLX as a novel strategy in the prevention of CTRCD.

In the pre-clinical setting, we have recently investigated the role of FLX and its bioactive components, ALA and SDG, in both the prevention and treatment of anthracycline mediated cardiotoxicity [20, 21, 25]. In 2020, using a chronic in vivo murine model of DOX + Trastuzuamb (DOX + TRZ)-mediated cardiotoxicity, mice were randomized to prophylactic treatment with dietary supplementation of 10% FLX, 4.4% ALA, or 0.44% SDG for a total of 6 weeks [20]. We demonstrated that FLX prevented adverse cardiovascular remodeling, mitigated myofibrillar disarray, and down-regulated inflammation, oxidative stress and mitochondrial dysfunction [20]. In a subsequent study, in 2022, we investigated whether the prophylactic administration of FLX was comparable to the RAS antagonist Perindopril (PER) in preventing CTRCD in a murine model [21]. Over a 6 week period, a total of 81 female mice were randomized to a 10% FLX-supplemented diet with or without PER prior to the administration of DOX + TRZ[21]. We demonstrated that pre-treatment with FLX was equivalent to ACEi in the prevention of LV systolic dysfunction by attenuating inflammatory oxylipins and modulation of the NF-KB signaling pathway [21]. Finally, in 2025, we investigated whether treatment with FLX and PER can reverse LV systolic dysfunction after the development of CRTCD [25]. In a chronic in vivo murine model, a total of 110 mice received DOX + TRZ for a total of 3 weeks, followed by daily consumption of 10% FLX, PER, or a combination of both agents for an additional 3 weeks [25]. Similar to our prevention studies[20, 21], we demonstrated that treatment with either FLX, PER or both FLX + PER improved LVEF, preserved myocyte integrity, and attenuated mitochondrial mediated ferroptosis, necrosis, and apoptosis, in mice with established DOX + TRZ mediated cardiotoxicity [25]. Collectively, these basic science studies validate the role of FLX in both the prevention and treatment of DOX + TRZ mediated cardiotoxicity.

With the aim of bridging the gap between our novel basic science findings and the clinical arena, the CANFLAX study investigated the cardioprotective role of FLX “milk” in women with breast cancer. All study participants completed their oat fibre “milk” or FLX “milk” intake by 4 months during concomitant treatment with an anthracycline based chemotherapy regimen [approximately 86% received 4 cycles of doxorubicin and cyclophosphamide (AC) therapy]. In our study population, we demonstrated that women randomized to FLX “milk” had a compliance rate of approximately 70% with a 2-fold increase in ALA plasma levels (Fig. 1), confirming ingestion of the dietary agent during treatment with anthracyclines. There was no change in the serial blood pressure, NT-proBNP levels, nor LVEF values in both the placebo oat fibre “milk” and the FLX “milk” groups; no study participant developed symptomatic CTRCD. However, the GLS parameters were reduced in the oat fibre “milk” group (−13.8 ± 0.3%) as compared to the FLX “milk” group (−18.4 ± 0.3%) at 4-months, during concomitant completion of their anthracycline based chemotherapy. Additionally, although the GLS at 6 months started to normalize at −15.1 ± 0.3% in the oat fibre “milk” group, it was still lower than the GLS at 6 months of −18.5 ± 0.2% for the FLX “milk” group (Table 2). As GLS is a sensitive non-invasive echocardiographic technique that allows for the early detection of LV systolic dysfunction, prior to a decrease in conventional LVEF parameters[26, 27], our findings from this small pilot RCT suggest that the consumption of FLX “milk” (30 g of FLX) may be cardioprotective in women receiving anthracyclines for their underlying breast cancer.

There are a few limitations in the CANFLAX study. First, this is a single-centre pilot study with a small sample size (*n* = 21) of women with breast cancer, none of whom developed CTRCD. A multi-centre study with a larger patient population, in whom at least 20% were to develop CRTCD, would provide additional statistical power to validate whether consumption of FLX “milk” is cardioprotective in this patient population. Second, although the majority received AC based chemotherapy in 86% of the study population, a total of 14% received FEC, which is another confounding variable in our small pilot RCT. Third, we did not control for natural variation in dietary intake of functional foods between the participants in both study groups which may be a confounding factor. Finally, as up to 30% of study participants reported GI related side effects from the intake of either the oat fibre “milk” or FLX “milk” products during their chemotherapy treatment, further efforts are required to improve the adherence rate of this natural food product.

## Conclusion

In women receiving an anthracycline based chemotherapy regimen for breast cancer, this small pilot study suggest that FLX “milk” consumption may prevent early cardiotoxicity as reflected by preservation of GLS parameters. Further evaluation in a large, multicentre trial with a higher proportion of patients developing CRTCD is required to evaluate the potential cardioprotective role of FLX in this patient population.

## Data Availability

All data generated or analysed during this study are included in this published article.
